# Exact and Numerical Solution of the Fractional Sturm–Liouville Problem with Neumann Boundary Conditions

**DOI:** 10.3390/e24020143

**Published:** 2022-01-18

**Authors:** Malgorzata Klimek, Mariusz Ciesielski, Tomasz Blaszczyk

**Affiliations:** 1 Department of Mathematics, Czestochowa University of Technology, al. Armii Krajowej 21, 42-200 Czestochowa, Poland; malgorzata.klimek@pcz.pl; 2 Department of Computer Science, Czestochowa University of Technology, Dabrowskiego 73, 42-200 Czestochowa, Poland; mariusz.ciesielski@icis.pcz.pl

**Keywords:** fractional Sturm–Liouville problem, Neumann boundary conditions, fractional calculus, numerical solution, integral equation

## Abstract

In this paper, we study the fractional Sturm–Liouville problem with homogeneous Neumann boundary conditions. We transform the differential problem to an equivalent integral one on a suitable function space. Next, we discretize the integral fractional Sturm–Liouville problem and discuss the orthogonality of eigenvectors. Finally, we present the numerical results for the considered problem obtained by utilizing the midpoint rectangular rule.

## 1. Introduction

The Sturm–Liouville boundary value problem (eigenvalue problem) is an important issue in the field of differential equations. These problems can be regular or singular at each endpoint of the considered interval. The classical Sturm–Liouville theory is still a branch of mathematics, where recent investigations yield meaningful results, both theoretical and applicable (compare [1,2] and references therein). Eigenvalue problems arise in a large number of disciplines of sciences and engineering (such as applied mathematics, classical physics or quantum mechanics).

Therefore, one of the most important problems, in the extended Sturm–Liouville theory, including fractional differential operators, is to understand how to to construct fractional analogues of a classical Sturm–Liouville operator and how its spectrum and eigenfunctions behave for various types of operator and boundary conditions (for example, for fractional Dirichlet, Neumann, Robin or mixed conditions).

It is well known from the classical spectral theory that the Dirichlet Laplacian on a bounded domain always has a purely discrete spectrum, while the Neumann Laplacian on a bounded domain may have an essential spectrum if the boundary is not smooth. For this reason, one can say that the Neumann eigenvalue problem is more subtle than the Dirichlet one [3].

This paper is a part of the project where we construct the fractional Sturm–Liouville Equation as an Euler-Lagrange Equation for a suitable action functional, including fractional derivatives. Here, we focus on the examination of the fractional Sturm–Liouville problem (FSLP) in a bounded interval [a,b] with homogeneous fractional Neumann boundary conditions. The problem is analyzed under assumption: 1/2<α≤1, where α is the order of fractional derivatives. Let us point out that by applying the fractional calculus and standard fractional derivatives, one can construct different types of the Sturm–Liouville operators and various types of boundary conditions (see, for example, results in papers [4,5,6,7,8,9,10,11,12]).

Fractional eigenvalue problems have also been considered within the framework of tempered fractional calculus [12,13] and conformable fractional calculus [14,15,16,17]. Recently, a fractional Sturm–Liouville operator containing composite fractional derivatives has been proposed in paper [18], and Prabhakar derivatives were applied in the construction of fractional eigenvalue problems subjected to homogeneous Dirichlet or mixed boundary conditions in papers [19,20].

However, it is important to be aware that not every construction of fractional eigenvalue problem leads to a purely discrete real spectrum of the FSLP or orthogonal eigenfunctions’ system, which is complete in the corresponding Hilbert space. For instance, fractional Sturm–Liouville operators proposed in papers [21,22,23,24,25,26,27], where numerical methods were developed to study eigenvalues and eigenfunctions, include only a left-sided fractional derivative/derivatives. Consequently, they do not lead to orthogonal systems of eigenfunctions, and in many cases, the spectrum includes a complex part. A similar remark applies to other models, where one-sided fractional derivatives or a mixture of classical and one-sided fractional derivatives were applied to develop rigorous analytical results for eigenvalue problems with Dirichlet or extended Dirichlet boundary conditions, dependent on the fractional order of derivatives [28,29,30,31]. Some results concerning discrete spectrum and orthogonal eigenfunction systems have been studied in papers [32,33], where an integer order part of the differential operator was supplemented with a composition or difference of the left and right fractional derivatives, respectively. Therefore, the motivation of our variational approach, first presented in papers [5,34], is to develop the formulation of a fractional Sturm–Liouville problem with an orthogonal system of eigenfunctions and real eigenvalues.

In this approach, the Sturm–Liouville operator contains both the left and right fractional derivatives [35,36], and equations containing this type of differential operators are known as the fractional Euler–Lagrange equations [37,38,39]. The application of this type of operator causes many difficulties in solving fractional differential equations. The same problem appears in calculating eigenvalues. It should be highlighted that for eigenvalue problems with differential operators, being a composition of the left and right fractional derivatives, only a few exact solutions are available [6,9,11,12,34]. For this reason, many scientists have been working on numerical methods dedicated to the fractional Euler-Lagrange equations [40,41,42,43,44] and/or the fractional Sturm–Liouville problems [45,46,47,48].

In our previous papers, we studied fractional eigenvalue problems with homogeneous Dirichlet [20,49,50], Neumann [51], Robin [52] and mixed [47,48] boundary conditions. In these papers, it was shown that the FSLP with the adequate boundary conditions has a purely discrete spectrum, and the corresponding system of eigenfunctions is orthogonal and complete in a suitable Hilbert space.

In the paper [48], we proposed the numerical method, based on the transformation of the FSLP, subjected to the homogeneous mixed boundary conditions into the equivalent integral eigenvalue problem. The integral operator with a kernel depending on both the form of the fractional differential operator and on boundary conditions was discretized to calculate approximate eigenvalues and eigenvectors. In addition, the presented approach gave us a possibility to approximate eigenfunctions, keeping the orthogonality of the eigenvectors and the approximated eigenfunctions at each step of the algorithm. We used the experimental rate of convergence to control the convergence of the developed numerical scheme, and we obtained a rate of convergence close to 2α.

In the present paper, the analogous technique is developed for FSLP with Neumann boundary conditions. However, when the homogeneous, fractional Neumann boundary conditions are applied on both boundaries of the domain, an additional integral constraint is necessary. The additional integral condition causes the development of a numerical scheme to become slightly more complex, as compared to the previous case (FSLP with mixed boundary conditions). In particular, two numerical schemes are studied—in the first, we use the same discretization procedure for both integrals, i.e., for an integral determining the operator and for an integral appearing in the definition of an integral constraint. In turn, in the second scheme, which we refer to as a hybrid numerical scheme, two different discretization procedures are allowed. The main results of the paper include two numerical schemes dedicated to FSLP with fractional Neumann boundary conditions, the results of the analysis of the eigenvectors’ orthogonality and examples of numerical solutions.

The paper is structured as follows: Section 2 deals with the fundamentals of FSLP theory. Section 3 describes two types of constructions of the discrete versions of integral FSLP. Section 4 presents results for the numerical solution of FSLP with homogeneous Neumann boundary conditions. Finally, Section 5 concludes the paper.

## 2. Preliminaries

Let us recall definitions of fractional operators, based on the following books [36,53]. First, the left and right Riemann–Liouville fractional derivatives are defined as:

(1) $$\begin{array}{ccc} D_{a^{+}}^{\alpha }\;y\left(x\right) & := & \frac{d}{dx}I_{a^{+}}^{1-\alpha }y\left(x\right), \end{array}$$

(2) $$\begin{array}{ccc} D_{b^{-}}^{\alpha }\;y\left(x\right) & := & -\frac{d}{dx}I_{b^{-}}^{1-\alpha }y\left(x\right), \end{array}$$

where integral operators $I_{a^{+}}^{\alpha }$ and $I_{b^{-}}^{\alpha }$ are the left and right fractional Riemann–Liouville integrals:

(3) $$\begin{array}{ccc} I_{a^{+}}^{\alpha }y\left(x\right) & := & \frac{1}{\Gamma \left(\alpha \right)}\int_{a}^{x}\frac{y\left(\tau \right)}{{\left(x-\tau \right)}^{1-\alpha }}d\tau ,\;x>a, \end{array}$$

(4) $$\begin{array}{ccc} I_{b^{-}}^{\alpha }y\left(x\right) & := & \frac{1}{\Gamma \left(\alpha \right)}\int_{x}^{b}\frac{y\left(\tau \right)}{{\left(\tau -x\right)}^{1-\alpha }}d\tau ,\;x<b. \end{array}$$

Then, the left and right Caputo fractional derivatives are defined as follows:

(5) $$\begin{array}{ccc} {}^{C}D_{a^{+}}^{\alpha }\;y\left(x\right) & := & D_{a^{+}}^{\alpha }\left(y\left(x\right)-y\left(a\right)\right), \end{array}$$

(6) $$\begin{array}{ccc} {}^{C}D_{b^{-}}^{\alpha }\;y\left(x\right) & := & D_{b^{-}}^{\alpha }\left(y\left(x\right)-y\left(b\right)\right). \end{array}$$

In this preliminary part of the paper, we shall report previous results enclosed in papers [34,51], relevant to developing a numerical method of solution of a fractional eigenvalue problem in the case when the solutions’ space is restricted by Neumann-type boundary conditions. Let us quote the general formulation of a regular fractional Sturm–Liouville problem (FSLP) introduced in [5,34].

**Definition** **1.** [Compare with Definition 5 in [34]**.** *Let α∈(0,1]. With the notation:*

(7) $$L_{q}:=D_{b-}^{\alpha }p\left(x\right)\;\;{}^{C}D_{a+}^{\alpha }+q\left(x\right),$$

*consider the fractional Sturm–Liouville Equation (FSLE):*

(8) $$L_{q}y_{\lambda }\left(x\right)=\lambda w\left(x\right)y_{\lambda }\left(x\right),$$

*where p(x)≠0,w(x)>0∀x∈[a,b], functions p,q,w are real-valued continuous functions in [a,b] and boundary conditions are:*

(9) $$\begin{array}{ccc} & c_{1}y_{\lambda }\left(a\right)+c_{2}I_{b-}^{1-\alpha }p\left(x\right){}^{C}D_{a+}^{\alpha }y_{\lambda }\left(x\right){|}_{x=a}=0, & \end{array}$$

(10) $$\begin{array}{ccc} & d_{1}y_{\lambda }\left(b\right)+d_{2}I_{b-}^{1-\alpha }p\left(x\right){}^{C}D_{a+}^{\alpha }y_{\lambda }\left(x\right){|}_{x=b}=0 & \end{array}$$

*with $c_{1}^{2}+c_{2}^{2}\neq 0$ and $d_{1}^{2}+d_{2}^{2}\neq 0.$ The problem of finding number λ (eigenvalue) such that the BVP has a non-trivial solution, $y_{\lambda }$ (eigenfunction) will be called the regular fractional Sturm–Liouville eigenvalue problem (FSLP).*

In [34], we proved that eigenvalues generated while solving the above Sturm–Liouville problem are real, and eigenfunctions associated to distinct eigenvalues are orthogonal with respect to the following scalar product:

$${\langle f,g\rangle }_{w}:=\int_{a}^{b}w\left(x\right)\bar{f(x)}g\left(x\right)dx.$$

Let us observe that for α=1, we recover the classical Sturm–Liouville problem (CSLP), where operator (7) becomes the second order differential operator, and Equation (8) is the classical Sturm–Liouville equation:

$$-\frac{d}{dx}p\left(x\right)\frac{dy_{\lambda }\left(x\right)}{dx}+q\left(x\right)y_{\lambda }\left(x\right)=\lambda w\left(x\right)y_{\lambda }\left(x\right)$$

with boundary conditions appearing as follows:

(11) $$c_{1}y_{\lambda }\left(a\right)+c_{2}\frac{dy_{\lambda }\left(a\right)}{dx}=0,\;d_{1}y_{\lambda }\left(b\right)+d_{2}\frac{dy_{\lambda }\left(b\right)}{dx}=0.$$

When we choose $c_{1}=d_{1}=0$ in Equation (11), determining the boundary conditions, we arrive at CSLP with homogeneous Neumann boundary conditions:

$$\frac{dy_{\lambda }\left(a\right)}{dx}=\frac{dy_{\lambda }\left(b\right)}{dx}=0.$$

The same choice of constants in the general fractional boundary conditions (9), (10) yields the fractional version of homogeneous Neumann boundary conditions. In this way, we restrict the general regular FSLP to the case with fractional homogeneous Neumann boundary conditions (14), (15), described in the definition below:

**Definition** **2.** *Let α∈(0,1]. With the following notation:*

(12) $$L_{q}:=D_{b-}^{\alpha }p\left(x\right)\;\;{}^{C}D_{a+}^{\alpha }+q\left(x\right),$$

*consider the fractional Sturm–Liouville equation:*

(13) $$L_{q}y_{\lambda }\left(x\right)=\lambda w\left(x\right)y_{\lambda }\left(x\right),$$

*where p(x)≠0,w(x)>0∀x∈[a,b], functions p,q,w are real-valued continuous functions in [a,b] and boundary conditions are:*

(14) $$\begin{array}{ccc} & I_{b-}^{1-\alpha }p\left(x\right){}^{C}D_{a+}^{\alpha }y_{\lambda }\left(x\right){|}_{x=a}=0, & \end{array}$$

(15) $$\begin{array}{ccc} & I_{b-}^{1-\alpha }p\left(x\right){}^{C}D_{a+}^{\alpha }y_{\lambda }\left(x\right){|}_{x=b}=0. & \end{array}$$

*In the problem of finding number λ (eigenvalue) such that the BVP has a non-trivial solution, $y_{\lambda }$ (eigenfunction) will be called the regular fractional Sturm–Liouville eigenvalue problem with homogeneous fractional Neumann boundary conditions (FSLPN).*

We focus the further investigations of numerical solutions to FSLPN, defined above, in the case when q=0, i.e., the fractional Sturm–Liouville operator (FSLO) is $L_{0}$:

(16) $$L_{0}:=D_{b-}^{\alpha }p\left(x\right)\;\;{}^{C}D_{a+}^{\alpha }$$

and we restrict fractional order to α∈(1/2,1]. These assumptions are motivated by the fact that, in such case, we can apply analytical results obtained in the paper [51]. These results are based on the transformation of the differential FSLPN into the equivalent integral problem. Moreover, they include theorems on purely discrete spectrum of differential and integral fractional Sturm–Liouville operators. The schedule of deriving the analytical spectral result for FSLPN contains the following preliminary steps: construction of the suitable solutions’ space, transformation to the integral FSLPN and proof of equivalence of both types of eigenvalue problems.

First, when we restrict our considerations to continuous solutions, we arrive at the following relation resulting from Equation (8) (for q=0) and composition properties of fractional derivatives and integrals:

(17) $$L_{0}\left[y_{\lambda }\left(x\right)-I_{a+}^{\alpha }\frac{1}{p(x)}I_{b-}^{\alpha }\lambda w\left(x\right)y_{\lambda }\left(x\right)\right]=0.$$

Similar to the case of FSLP subjected to the homogeneous mixed, Dirichlet, and Robin boundary conditions [20,47,48,52], we note that while assuming the continuity of $y_{\lambda }$ and 1≥α>1/2, this eigenfunction fulfills the following integral Equation of interval [a,b]:

(18) $$y_{\lambda }\left(x\right)-I_{a+}^{\alpha }\frac{1}{p(x)}I_{b-}^{\alpha }\lambda w\left(x\right)y_{\lambda }\left(x\right)=A_{1}+A_{2}I_{a+}^{\alpha }\frac{{\left(b-x\right)}^{\alpha -1}}{p(x)},$$

where the function on the right-hand side of Equation (18) is an arbitrary continuous function from the kernel of operator $L_{0}$. Therefore, in fact, we work on the subspace of continuous functions, defined previously in [51] and described in the definition below.

**Definition** **3.** *Let $C_{p}\left[a,b\right]\subset C\left[a,b\right]$ be the subspace of continuous functions given as follows:*

(19) $$f\in C_{p}\left[a,b\right]\Leftrightarrow f\left(x\right)=I_{a+}^{\alpha }\frac{1}{p(x)}I_{b-}^{\alpha }\psi \left(x\right)+A_{1}+A_{2}I_{a+}^{\alpha }\frac{{\left(b-x\right)}^{\alpha -1}}{p(x)},$$

*where x∈[a,b], ψ∈C[a,b], and $A_{1},A_{2}$ are constants.*

On the considered function space, $f\left(a\right)=A_{1}$, therefore, the fractional Caputo derivative ${}^{C}D_{a+}^{\alpha }f$ exists for every point in [a,b). Now, we restrict the defined subspace to functions fulfilling the homogeneous fractional Neumann boundary conditions. In addition, an integral condition results from initial FSLE (8) and assumed boundary conditions. This property of the solutions’ space is also necessary in the classical case α=1.

**Definition** **4.** *Let $C_{p,N}\left[a,b\right]\subset C_{p}\left[a,b\right]$ be the subspace of continuous functions given in Definition 3, fulfilling homogeneous, fractional Neumann boundary conditions (14), (15) and the integral condition that:*

(20) $$\int_{a}^{b}w\left(x\right)f\left(x\right)dx=0.$$

It is easy to verify that constants $A_{1}$ and $A_{2}$ in Equation (18) are determined by the homogeneous fractional Neumann boundary conditions (14), (15) and condition (20). Namely, we have:

(21) $$A_{1}=-\lambda \frac{\int_{a}^{b}w\left(x\right)\left(I_{a+}^{\alpha }\frac{1}{p(x)}I_{b-}^{\alpha }w\left(x\right)y_{\lambda }\left(x\right)\right)dx}{\int_{a}^{b}w\left(\xi \right)y_{\lambda }\left(\xi \right)d\xi },$$

(22) $$A_{2}=0.$$

Thence, we obtain the integral Equation (18) in the exact form:

(23) $$y_{\lambda }\left(x\right)=\lambda T_{w}y_{\lambda }\left(x\right):=\lambda I_{a+}^{\alpha }\frac{1}{p(x)}I_{b-}^{\alpha }w\left(x\right)y_{\lambda }\left(x\right)$$

$$-\lambda \frac{\int_{a}^{b}w\left(x\right)\left(I_{a+}^{\alpha }\frac{1}{p(x)}I_{b-}^{\alpha }w\left(x\right)y_{\lambda }\left(x\right)\right)dx}{\int_{a}^{b}w\left(\xi \right)y_{\lambda }\left(\xi \right)d\xi }.$$

The integral operator $T_{w}$, given above as the composition of the left and right Riemann–Liouville integrals, can be expressed as an integral operator with kernel $K_{w}$ [51]:

(24) $$y_{\lambda }\left(x\right)=\lambda T_{w}y_{\lambda }\left(x\right)=\lambda \int_{a}^{b}K_{w}\left(x,s\right)y_{\lambda }\left(s\right)ds,$$

where kernel $K_{w}$ is given below:

(25) $$K_{w}\left(x,s\right)=w\left(s\right)\left(K_{1}\left(x,s\right)-\frac{\int_{a}^{b}w\left(\xi \right)K_{1}\left(\xi ,s\right)d\xi }{\int_{a}^{b}w\left(\xi \right)d\xi }\right)$$

and symmetric kernel part $K_{1}$ is of the form:

(26) $$K_{1}\left(x,s\right)=\frac{1}{\Gamma^{2}\left(\alpha \right)}\int_{a}^{\min\left(x,s\right)}\frac{{\left(x-t\right)}^{\alpha -1}{\left(s-t\right)}^{\alpha -1}}{p\left(t\right)}dt.$$

The above explicit expressions for kernels $K_{1}$ and $K_{w}$ result from the application of the integration rule-change of the order of integration. We note that kernel $K_{w}$, given in Equation (25), fulfills the condition:

(27) $$\int_{a}^{b}K_{w}\left(x,s\right)w\left(x\right)dx=0$$

which yields the properties:

(28) $$f\in C_{p,N}\left[a,b\right]⟹T_{w}f\in C_{p,N}\left[a,b\right]$$

and

(29) $$f\in \tilde{L_{w}^{2}}\left(a,b\right)⟹T_{w}f\in C_{p,N}\left[a,b\right],$$

where Hilbert space $\tilde{L_{w}^{2}}\left(a,b\right)$ is defined by Equation (33).

We start proving the above properties by observation that in the case when the weight function w∈C[a,b] and the fractional order fulfills α∈(1/2,1] we have for any function $f\in L_{w}^{2}\left(a,b\right)$ that the results of fractional integration are continuous functions, i.e., $I_{a+}^{\alpha }f\in C\left[a,b\right]$ and $I_{b-}^{\alpha }f\in C\left[a,b\right]$. Then, we apply composition rules for fractional operators [36,51] and Formula (23) to check Neumann boundary conditions (14), (15) for the respective images of continuous or Lebegue functions:

$$I_{b-}^{1-\alpha }p\left(x\right){}^{C}D_{a+}^{\alpha }T_{w}f\left(x\right){|}_{x=a}=$$

$$=I_{b-}^{1-\alpha }p\left(x\right){}^{C}D_{a+}^{\alpha }\left(I_{a+}^{\alpha }\frac{1}{p(x)}I_{b-}^{\alpha }w\left(x\right)f\left(x\right)-\frac{\int_{a}^{b}w\left(x\right)\left(I_{a+}^{\alpha }\frac{1}{p(x)}I_{b-}^{\alpha }w\left(x\right)f\left(x\right)\right)dx}{\int_{a}^{b}w\left(\xi \right)y_{\lambda }\left(\xi \right)d\xi }\right){|}_{x=a}$$

$$=I_{b-}^{1}w\left(x\right)f\left(x\right)dx{|}_{x=a}=\int_{a}^{b}w\left(x\right)f\left(x\right)dx=0$$

because, by assumption, function $f\in C_{p,N}\left[a,b\right]$ or $f\in \tilde{L_{w}^{2}}\left(a,b\right)$. Similarly, we obtain at the endpoint of the interval for continuous or Lebegue function *f* the following result:

$$I_{b-}^{1-\alpha }p\left(x\right){}^{C}D_{a+}^{\alpha }T_{w}f\left(x\right){|}_{x=b}=$$

$$=I_{b-}^{1-\alpha }p\left(x\right){}^{C}D_{a+}^{\alpha }\left(I_{a+}^{\alpha }\frac{1}{p(x)}I_{b-}^{\alpha }w\left(x\right)f\left(x\right)-\frac{\int_{a}^{b}w\left(x\right)\left(I_{a+}^{\alpha }\frac{1}{p(x)}I_{b-}^{\alpha }w\left(x\right)f\left(x\right)\right)dx}{\int_{a}^{b}w\left(\xi \right)y_{\lambda }\left(\xi \right)d\xi }\right){|}_{x=b}$$

$$=I_{b-}^{1-\alpha }\left(I_{b-}^{\alpha }w\left(x\right)f\left(x\right)\right){|}_{x=b}=0$$

as for order α∈(1/2,1] and w∈C[a,b] we have $I_{b-}^{\alpha }wf\in C\left[a,b\right]$.

In summary, we observe that images $T_{w}f$ fulfill Neumann boundary conditions (14), (15) for any function from $C_{p,N}$ or $\tilde{L_{w}^{2}}\left(a,b\right)$ spaces. Now, let us check the integral condition (20) for image $T_{w}f$. We apply kernel property (27) and obtain for arbitrary $f\in C_{p,N}$ or $f\in \tilde{L_{w}^{2}}\left(a,b\right)$ the following integral formula after the change in the order of integration:

$$\int_{a}^{b}w\left(x\right)T_{w}f\left(x\right)dx=\int_{a}^{b}w\left(x\right)\left(\int_{a}^{b}K_{w}\left(x,s\right)f\left(s\right)ds\right)dx$$

$$=\int_{a}^{b}f\left(s\right)\left(\int_{a}^{b}K_{w}\left(x,s\right)w\left(x\right)dx\right)ds=0.$$

The above calculations show that properties (28), (29) are valid.

The following two lemmas lead to the main result on spectral properties of the considered FSLPN. The first one says that on the constructed solutions’ space operator, $T_{w}$ is an inverse operator to the fractional Sturm–Liouville operator ${\frac{1}{w}L}_{0}$, and it results from work performed in the previous paper [51] (compare relations in Equations (23) and (24), and the corresponding calculations).

**Lemma** **1.** *Let $1\geq \alpha >1/2,\frac{1}{p},w\;\in C\left[a,b\right]$ and $f\in C_{p,N}\left[a,b\right]$. Then, the following relations are valid for any x∈[a,b]:*

(30) $$T_{w}\frac{1}{w(x)}L_{0}f\left(x\right)=f\left(x\right),$$

(31) $$\frac{1}{w(x)}L_{0}T_{w}f\left(x\right)=f\left(x\right).$$

The next lemma on the equivalence of the integral and differential FSLPN is a straightforward corollary of Equations (30) and (31).

**Lemma** **2.** [Compare Lemma 1 in [51]]**.** *Let $1\geq \alpha >1/2,\frac{1}{p},w\;\in C\left[a,b\right]$ and $f\in C_{p,N}\left[a,b\right]$.*

*Then, the following equivalence is valid:*

(32) $$L_{0}f\left(x\right)=\lambda w\left(x\right)f\left(x\right)⟺T_{w}f\left(x\right)=\frac{1}{\lambda }f\left(x\right).$$

In the paper [51], we studied properties of the $T_{w}$ operator and derived the following result on its spectrum. We denote the Hilbert space $\tilde{L_{w}^{2}}\left(a,b\right)$ as follows:

(33) $$\tilde{L_{w}^{2}}\left(a,b\right):=\left\{f\in L_{w}^{2}\left(a,b\right);\;\;\;\int_{a}^{b}w\left(x\right)f\left(x\right)dx=0\right\}$$

which means that it is a subspace of $L_{w}^{2}\left(a,b\right)$ containing functions fulfilling condition (20). Now, let us quote the theorem on the spectrum of integral and differential FSLP with Neumann boundary conditions (14), (15).

**Theorem** **1.** *Let $1\geq \alpha >\frac{1}{2}$ and $\frac{1}{p},\;w\in C\left[a,b\right]$. Then, operator $T_{w}$, given in (23), (24), has a purely discrete spectrum enclosed in interval (−1,1), with 0 being its only limit point. Eigenfunctions $y_{n}$ corresponding to the respective eigenvalues belong to the $C_{p,N}\left[a,b\right]$-space and form a basis in the $\tilde{L_{w}^{2}}\left(a,b\right)$-space (33).*

Finally, we recall that the above theorem, together with Lemma 2, yields the result on the spectrum and eigenfunctions for the differential FSLP with homogeneous Neumann type boundary conditions.

**Theorem** **2.** *Let $1\geq \alpha >\frac{1}{2}$ and $\frac{1}{p},\;w\in C\left[a,b\right]$. Then, operator $L_{0}$, given in (16), considered on the $C_{p,N}\left[a,b\right]$-space, has a purely discrete real spectrum with $|\lambda_{n}|\to \infty$. Eigenfunctions $y_{n}$ form a basis in the $\tilde{L_{w}^{2}}\left(a,b\right)$-space (33). For positive function p>0, the spectrum is positive, whereas for negative p<0, it is negative. Moreover, the following number series is convergent:*

(34) $$\sum_{n=1}^{\infty }\frac{1}{{\left(\lambda_{n}\right)}^{2}}<\infty$$

*and the inequality below is fulfilled for certain $M_{+}>0$:*

(35) $$\frac{|y_{n}\left(x\right)|}{|\lambda_{n}|}\leq M_{+},\;x\in \left[a,b\right],\;n\in N.$$

Having summarized the approach, developed in [51], to study properties and the spectrum of FSLO (16) on function space subject to the homogeneous fractional Neumann boundary conditions, we are now ready to present the numerical methods of solution to FSLPN.

## 3. Main Results

In this section, two approaches to the construction of the discrete version of integral FSLPN are presented. We discuss an influence of each of the proposed methods on the ortogonality property of eigenvectors of discrete integral operator.

### 3.1. Case I—One Numerical Integration Scheme in the Construction of Discrete Integral FSLPN

Let us observe that passing to the discrete form of the integral eigenvalue problem (24), we construct discrete versions of integrals describing the $T_{w}$-operator itself (24), its kernel (25) and its integral condition (20). First, we shall consider the case when we use the same numerical procedure for integrals in (20) and (24). We divide the interval [a,b] into *N* subintervals and choose arbitrary points $x_{i},i=1,$ …$,N^{\prime }$ according to the applied rule of numerical integration. In particular, for an equidistant partition into *N* subintervals, we have $N^{\prime }=N$ for rectangular approximation, $N^{\prime }=N+1$ for the trapezoid method while Simpson’s method requires $N^{\prime }=N+1$ (*N* must be even). In general, the numerical integral is given as:

(36) $$\int_{a}^{b}y\left(x\right)dx\approx \sum_{i=1}^{N^{\prime }}\beta_{i}y\left(x_{i}\right)$$

and the $T_{w}$-integral operator acts as follows:

(37) $$T_{w}y\left(x_{i}\right)=\sum_{j=1}^{N^{\prime }}\beta_{j}K_{w}\left(x_{i},x_{j}\right)y\left(x_{j}\right),$$

where approximation weights $\beta_{i},\;\;i=1,$ …$,N^{\prime }$ depend on the specific choice of points $x_{i},\;\;i=1,$ …$,N^{\prime }$ corresponding to the applied method of numerical integration. Denoting values of function $y\left(x_{i}\right)=Y_{i}$ and vector $Y=(Y_{1},Y_{2},$ …$,Y_{N^{\prime }}{)}^{T}$, we rewrite the above Equation to the discrete form, where operator $T_{w}$ acts on vectors from $N^{\prime }$-dimensional vector space:

(38) $${\left(T_{w}Y\right)}_{i}=\sum_{j=1}^{N^{\prime }}\beta_{j}K_{w}\left(x_{i},x_{j}\right)Y_{j}$$

subjected to the discrete version of condition (20):

(39) $$\sum_{i=1}^{N^{\prime }}\beta_{i}w_{i}Y_{i}=0.$$

At this point, we write the discrete version of the kernel, assuming that the integrals on the right-hand side of equality (27) are discretized according to Equation (36):

(40) $$K_{w}\left(x_{i},x_{j}\right)=w_{j}K_{1}\left(x_{i},x_{j}\right)-\frac{w_{j}\sum_{k=1}^{N^{\prime }}\beta_{k}w_{k}K_{1}\left(x_{k},x_{j}\right)}{\sum_{l=1}^{N^{\prime }}\beta_{l}w_{l}}.$$

We note that the discrete analog of the kernel property (27) is fulfilled:

(41) $$\sum_{i=1}^{N^{\prime }}\beta_{i}w_{i}K_{w}\left(x_{i},x_{j}\right)=0$$

and that the image of the vector fulfilling the condition (39) obeys the same condition:

(42) $$\sum_{i=1}^{N^{\prime }}\beta_{i}w_{i}{\left(T_{w}Y\right)}_{i}=0.$$

This property of the discrete integral operator $T_{w}$ can be easily checked:

$$\sum_{i=1}^{N^{\prime }}\beta_{i}w_{i}{\left(T_{w}Y\right)}_{i}=\sum_{i=1}^{N^{\prime }}\beta_{i}w_{i}\left(\sum_{j=1}^{N^{\prime }}\beta_{j}K_{w}\left(x_{i},x_{j}\right)Y_{j}\right)$$

$$=\sum_{j=1}^{N^{\prime }}\beta_{j}Y_{j}\left(\sum_{i=1}^{N^{\prime }}\beta_{i}w_{i}K_{w}\left(x_{i},x_{j}\right)\right)=0.$$

In conclusion, we consider the discrete eigenvalue problem:

(43) $$Y_{\lambda }=\lambda T_{w}Y_{\lambda },\;\;\;\;\;\;{\left(Y_{\lambda }\right)}_{i}=\lambda \sum_{j=1}^{N^{\prime }}\beta_{j}K_{w}\left(x_{i},x_{j}\right){\left(Y_{\lambda }\right)}_{j}$$

on the $N^{\prime }$-dimensional space of vectors fulfilling condition (39). In the next step, we shall study the presented discrete eigenvalue problem, where all the discrete analogs of integrals from the fractional integral eigenvalue problem are constructed by using the same numerical integration method. In addition, the scalar product on the vector space is also defined according to the discretization procedure (36) and weight *w*:

(44) $${\langle F,G\rangle }_{w}:=\sum_{i=1}^{N^{\prime }}\beta_{i}w_{i}F_{i}G_{i}.$$

The following lemma describes the orthogonality property of eigenvectors of discrete operator $T_{w}$.

**Lemma** **3.** *Eigenvectors corresponding to distinct eigenvalues of the discrete FSLP (43) are orthogonal with respect to scalar product (44), i.e.,*

(45) $${\langle Y_{\lambda },Y_{\rho }\rangle }_{w}=0\;\lambda \neq \rho .$$

**Proof.**  In the proof, we apply condition (39) and the symmetricity of kernel part $K_{1}$:

$$\frac{1}{\rho }{\langle Y_{\lambda },Y_{\rho }\rangle }_{w}={\langle Y_{\lambda },T_{w}Y_{\rho }\rangle }_{w}=\sum_{i=1}^{N^{\prime }}\beta_{i}w_{i}{\left(Y_{\lambda }\right)}_{i}{\left(T_{w}Y_{\rho }\right)}_{i}=$$

$$=\sum_{i=1}^{N^{\prime }}\beta_{i}w_{i}{\left(Y_{\lambda }\right)}_{i}\left(\sum_{j=1}^{N^{\prime }}\beta_{j}K_{w}\left(x_{i},x_{j}\right){\left(Y_{\rho }\right)}_{j}\right)$$

$$=\sum_{i=1}^{N^{\prime }}\beta_{i}w_{i}{\left(Y_{\lambda }\right)}_{i}\left[\sum_{j=1}^{N^{\prime }}\beta_{j}w_{j}{\left(Y_{\rho }\right)}_{j}\left(K_{1}\left(x_{i},x_{j}\right)-\frac{\sum_{k=1}^{N^{\prime }}\beta_{k}w_{k}K_{1}\left(x_{k},x_{j}\right)}{\sum_{l=1}^{N^{\prime }}\beta_{l}w_{l}}\right)\right]$$

$$=\sum_{j=1}^{N^{\prime }}\sum_{i=1}^{N^{\prime }}\beta_{i}w_{i}{\left(Y_{\lambda }\right)}_{i}\beta_{j}w_{j}{\left(Y_{\rho }\right)}_{j}K_{1}\left(x_{i},x_{j}\right)$$

$$=\sum_{j=1}^{N^{\prime }}\beta_{j}w_{j}{\left(Y_{\rho }\right)}_{j}\left(\sum_{i=1}^{N^{\prime }}\beta_{i}w_{i}{\left(Y_{\lambda }\right)}_{i}K_{1}\left(x_{j},x_{i}\right)\right)$$

$$=\sum_{j=1}^{N^{\prime }}\beta_{j}w_{j}{\left(Y_{\rho }\right)}_{j}\left[\sum_{i=1}^{N^{\prime }}\beta_{i}{\left(Y_{\lambda }\right)}_{i}\left(w_{i}K_{1}\left(x_{j},x_{i}\right)-\frac{w_{i}\sum_{k=1}^{N^{\prime }}\beta_{k}w_{k}K_{1}\left(x_{k},x_{i}\right)}{\sum_{l=1}^{N^{\prime }}\beta_{l}w_{l}}\right)\right]$$

$$=\sum_{j=1}^{N^{\prime }}\beta_{j}w_{j}{\left(Y_{\rho }\right)}_{j}\left(\sum_{i=1}^{N^{\prime }}\beta_{i}{\left(Y_{\lambda }\right)}_{i}K_{w}\left(x_{j},x_{i}\right)\right)$$

$$=\sum_{j=1}^{N^{\prime }}\beta_{j}w_{j}{\left(Y_{\rho }\right)}_{j}{\left(T_{w}Y_{\lambda }\right)}_{j}={\langle Y_{\rho },T_{w}Y_{\lambda }\rangle }_{w}=\frac{1}{\lambda }{\langle Y_{\lambda },Y_{\rho }\rangle }_{w}.$$

Finally, we obtain:

$$\left(\frac{1}{\rho }-\frac{1}{\lambda }\right){\langle Y_{\lambda },Y_{\rho }\rangle }_{w}=0$$

and as the eigenvalues are distinct, we end the proof:

$${\langle Y_{\lambda },Y_{\rho }\rangle }_{w}=0.$$

□

### 3.2. Case II—Hybrid Numerical Integration Scheme in the Construction of Discrete Integral FSLPN

Now, we shall investigate an influence of introducing two numerical integration schemes in the construction of the discrete version of integral FSLPN. Again, we divide the interval into *N* subintervals and choose points $S_{1}=\{{\tilde{x}}_{1},$ …$,{\tilde{x}}_{N_{1}}\}$ according to the rule of numerical integration which will be applied in the construction of the $T_{w}$ operator and the set of points (some may coincide with the previous ones) $S_{2}=\{{\bar{x}}_{1},$ …$,{\bar{x}}_{N_{2}}\}$ corresponding to numerical integration for kernel (25). In addition, we assume $S_{1}\subseteq S_{2}$ and denote the respective weights as $\tilde{\beta }=({\tilde{\beta }}_{1},$ …$,{\tilde{\beta }}_{N_{1}})$ and $\bar{\gamma }=({\bar{\gamma }}_{1},$ …$,{\bar{\gamma }}_{N_{2}})$. Then, the numerical versions of integral over interval [a,b] are:

(46) $$\int_{a}^{b}y\left(x\right)dx\approx \sum_{i=1}^{N_{1}}{\tilde{\beta }}_{i}y\left({\tilde{x}}_{i}\right),$$

(47) $$\int_{a}^{b}y\left(x\right)dx\approx \sum_{i=1}^{N_{2}}{\bar{\gamma }}_{i}y\left({\bar{x}}_{i}\right).$$

The discrete form of integral FSLP looks as follows:

(48) $$y\left({\bar{x}}_{i}\right)=\lambda T_{w}y\left({\bar{x}}_{i}\right)\;T_{w}y\left({\bar{x}}_{i}\right)=\sum_{j=1}^{N_{1}}{\tilde{\beta }}_{j}K_{w}\left({\bar{x}}_{i},{\tilde{x}}_{j}\right)y\left({\tilde{x}}_{j}\right),\;i=1,\cdots ,N_{2}$$

where kernel is (i=1, …$,N_{2}$ and j=1, …$,N_{1}$)

(49) $$K_{w}\left({\bar{x}}_{i},{\tilde{x}}_{j}\right)={\tilde{w}}_{j}K_{1}\left({\bar{x}}_{i},{\tilde{x}}_{j}\right)-\frac{{\tilde{w}}_{j}\sum_{k=1}^{N_{2}}{\bar{\gamma }}_{k}{\bar{w}}_{k}K_{1}\left({\bar{x}}_{k},{\tilde{x}}_{j}\right)}{\sum_{l=1}^{N_{2}}{\bar{\gamma }}_{l}{\bar{w}}_{l}}.$$

In the case $S_{1}=S_{2}$, we have $N_{1}=N_{2}$. Therefore, the matrix of operator $T_{w}$ is a quadratic one, and set (48) simultaneously yields the eigenvalues and eigenvectors of the discrete version of the integral FSLPN. In turn, when we work under assumption $S_{1}\subset S_{2}$, we note that the matrix of operator $T_{w}$ is a rectangular one, and the set of Equation (48) should be split into two parts. The first is:

(50) $$y\left({\bar{x}}_{i}\right)=\lambda T_{w}y\left({\bar{x}}_{i}\right)\;T_{w}y\left({\bar{x}}_{i}\right)=\sum_{j=1}^{N_{1}}{\tilde{\beta }}_{j}K_{w}\left({\bar{x}}_{i},{\tilde{x}}_{j}\right)y\left({\tilde{x}}_{j}\right),\;i=1,\cdots ,N_{1}$$

and we call it a reduced discrete integral FSLPN. It provides $N_{1}$ non-zero eigenvalues of discrete integral FSLPN and $N_{1}$ eigenvectors up to the first $N_{1}$ coordinates. The remaining equations of set (48) allow us to calculate eigenvector coordinates for $i=N_{1}+1,$ …$,N_{2}$. We reformulate numerical integration schemes by joining all the chosen points in set $S=S_{1}\cup S_{2}=S_{2}$, which means:

$$x_{i}={\tilde{x}}_{i}={\bar{x}}_{i}\;\;\;\;\;x_{i}\in S_{1}\;\wedge \;\;\;\;\;x_{i}={\tilde{x}}_{i}\;\;\;\;\;\;x_{i}\notin S_{1}.$$

Then, we construct the respective extended weights as follows:

$$\beta_{i}={\bar{\beta }}_{i}\;\;\;\;x_{i}\in S_{1}\;\wedge \;\;\;\beta_{i}=0\;\;\;\;\;x_{i}\notin S_{1},$$

$$\gamma_{i}={\tilde{\gamma }}_{i}\;\;\;\;x_{i}\in S_{2}\;\wedge \;\;\;\gamma_{i}=0\;\;\;\;\;x_{i}\notin S_{2}.$$

In general, the numerical integration rules (46) and (47) now become:

(51) $$\int_{a}^{b}y\left(x\right)dx\approx \sum_{i=1}^{N_{1}}{\tilde{\beta }}_{i}y\left({\tilde{x}}_{i}\right)=\sum_{i=1}^{N^{\prime }}\beta_{i}y\left(x_{i}\right),$$

(52) $$\int_{a}^{b}y\left(x\right)dx\approx \sum_{i=1}^{N_{2}}{\bar{\gamma }}_{i}y\left({\bar{x}}_{i}\right)=\sum_{i=1}^{N^{\prime }}\gamma_{i}y\left(x_{i}\right).$$

The $T_{w}$-integral operator, rewritten by using numerical integration rule (51), is:

(53) $$T_{w}y\left(x_{i}\right)=\sum_{j=1}^{N^{\prime }}\beta_{j}K_{w}\left(x_{i},x_{j}\right)y\left(x_{j}\right),$$

where weights $\beta_{i},\;\;i=1,$ …$,N^{\prime }$ are described above. The discrete extended version of kernel appears as follows:

(54) $$K_{w}\left(x_{i},x_{j}\right)=w_{j}K_{1}\left(x_{i},x_{j}\right)-\frac{w_{j}\sum_{k=1}^{N^{\prime }}\gamma_{k}w_{k}K_{1}\left(x_{k},x_{j}\right)}{\sum_{l=1}^{N^{\prime }}\gamma_{l}w_{l}},$$

where we assume that the integrals on the right-hand side of equality (25) are discretized according to rule (52). Similar to the previous scheme, the discrete analog of kernel property (27) is fulfilled:

(55) $$\sum_{i=1}^{N^{\prime }}\gamma_{i}w_{i}K_{w}\left(x_{i},x_{j}\right)=0\;j=1,\cdots ,N^{\prime }.$$

This property of kernel leads to the $N^{\prime }$-dimensional vector space of solutions obeying the numerical version of condition (20) in the form of:

(56) $$\sum_{i=1}^{N^{\prime }}\gamma_{i}w_{i}Y_{i}=0.$$

Similar to the scheme discussed previously, the image of the vector obeying condition (56) obeys the same condition:

(57) $$\sum_{i=1}^{N^{\prime }}\gamma_{i}w_{i}{\left(T_{w}Y\right)}_{i}=0$$

and this property of the discrete integral operator $T_{w}$ is a straightforward corollary of property (55):

$$\sum_{i=1}^{N^{\prime }}\gamma_{i}w_{i}{\left(T_{w}Y\right)}_{i}=\sum_{i=1}^{N^{\prime }}\gamma_{i}w_{i}\left(\sum_{j=1}^{N^{\prime }}\beta_{j}K_{w}\left(x_{i},x_{j}\right)Y_{j}\right)$$

$$=\sum_{j=1}^{N^{\prime }}\beta_{j}Y_{j}\left(\sum_{i=1}^{N^{\prime }}\gamma_{i}w_{i}K_{w}\left(x_{i},x_{j}\right)\right)=0.$$

In conclusion, in this section, we consider some properties of solutions of discrete FSLPN:

(58) $$Y_{\lambda }=\lambda T_{w}Y_{\lambda },\;\;\;\;\;\;{\left(Y_{\lambda }\right)}_{i}=\lambda \sum_{j=1}^{N^{\prime }}\beta_{j}K_{w}\left(x_{i},x_{j}\right){\left(Y_{\lambda }\right)}_{j}$$

on the $N^{\prime }$-dimensional space of vectors fulfilling condition (56), equipped with the scalar product defined according to the numerical integration rule (52):

(59) $${\langle F,G\rangle }_{w}:=\sum_{i=1}^{N^{\prime }}\gamma_{i}w_{i}F_{i}G_{i}.$$

The following lemma describes the control of orthogonality breaking for the eigenvectors of discrete operator $T_{w}$.

**Lemma** **4.** *The scalar product (59) of eigenvectors corresponding to distinct non-zero eigenvalues obeys the following inequality:*

(60) $$\left|\left(\frac{1}{\rho }-\frac{1}{\lambda }\right){\langle Y_{\lambda },Y_{\rho }\rangle }_{w}\right|\leq 2||w||\sqrt{\sum_{j=1}^{N^{\prime }}\beta_{j}^{2}}\sqrt{\sum_{j=1}^{N^{\prime }}{\left(M_{err,j}\right)}^{2}},$$

*where we denote errors of numerical integration generated by the respective sets of weights β,γ:*

$$\begin{array}{ccc} & Err_{\beta }\left(y_{\lambda }^{ap}\left(\cdot \right)K_{w}\left(x_{j},\cdot \right)\right)=\left|\sum_{i=1}^{N^{\prime }}\beta_{i}{\left(Y_{\lambda }\right)}_{i}K_{w}\left(x_{j},x_{i}\right)-\int_{a}^{b}y_{\lambda }^{ap}\left(x\right)K_{w}\left(x_{j},x\right)dx\right|, & \\ & Err_{\gamma }\left(y_{\lambda }^{ap}\left(\cdot \right)K_{w}\left(x_{j},\cdot \right)\right)=\left|\sum_{i=1}^{N^{\prime }}\gamma_{i}{\left(Y_{\lambda }\right)}_{i}K_{w}\left(x_{j},x_{i}\right)-\int_{a}^{b}y_{\lambda }^{ap}\left(x\right)K_{w}\left(x_{j},x\right)dx\right|, & \end{array}$$

*with $y_{\lambda }^{ap}$ being an approximate eigenfunction constructed by using the coordinates of eigenvector $Y_{\lambda }$ and*

(61) $$M_{err,j}:=\max_{\beta ,\gamma }\left\{Err_{\beta }\left(y_{\lambda }^{ap}\left(\cdot \right)K_{w}\left(x_{j},\cdot \right)\right),Err_{\gamma }\left(y_{\lambda }^{ap}\left(\cdot \right)K_{w}\left(x_{j},\cdot \right)\right)\right\}.$$

**Proof.**  In the first part of the proof, we apply condition (56) and the symmetricity of kernel $K_{1}$:

$$\frac{1}{\rho }{\langle Y_{\lambda },Y_{\rho }\rangle }_{w}={\langle Y_{\lambda },T_{w}Y_{\rho }\rangle }_{w}=\sum_{i=1}^{N^{\prime }}\gamma_{i}w_{i}{\left(Y_{\lambda }\right)}_{i}{\left(T_{w}Y_{\rho }\right)}_{i}=$$

$$=\sum_{i=1}^{N^{\prime }}\gamma_{i}w_{i}{\left(Y_{\lambda }\right)}_{i}\left(\sum_{j=1}^{N^{\prime }}\beta_{j}K_{w}\left(x_{i},x_{j}\right){\left(Y_{\rho }\right)}_{j}\right)$$

$$=\sum_{i=1}^{N^{\prime }}\gamma_{i}w_{i}{\left(Y_{\lambda }\right)}_{i}\left[\sum_{j=1}^{N^{\prime }}\beta_{j}w_{j}{\left(Y_{\rho }\right)}_{j}\left(K_{1}\left(x_{i},x_{j}\right)-\frac{\sum_{k=1}^{N^{\prime }}\gamma_{k}w_{k}K_{1}\left(x_{k},x_{j}\right)}{\sum_{l=1}^{N^{\prime }}\gamma_{l}w_{l}}\right)\right]$$

$$=\sum_{j=1}^{N^{\prime }}\sum_{i=1}^{N^{\prime }}\gamma_{i}w_{i}{\left(Y_{\lambda }\right)}_{i}\beta_{j}w_{j}{\left(Y_{\rho }\right)}_{j}K_{1}\left(x_{i},x_{j}\right)$$

$$=\sum_{j=1}^{N^{\prime }}\beta_{j}w_{j}{\left(Y_{\rho }\right)}_{j}\left(\sum_{i=1}^{N^{\prime }}\gamma_{i}w_{i}{\left(Y_{\lambda }\right)}_{i}K_{1}\left(x_{j},x_{i}\right)\right)$$

$$=\sum_{j=1}^{N^{\prime }}\beta_{j}w_{j}{\left(Y_{\rho }\right)}_{j}\left[\sum_{i=1}^{N^{\prime }}\gamma_{i}{\left(Y_{\lambda }\right)}_{i}\left(w_{i}K_{1}\left(x_{j},x_{i}\right)-\frac{w_{i}\sum_{k=1}^{N^{\prime }}\gamma_{k}w_{k}K_{1}\left(x_{k},x_{i}\right)}{\sum_{l=1}^{N^{\prime }}\gamma_{l}w_{l}}\right)\right]$$

$$=\sum_{j=1}^{N^{\prime }}\beta_{j}w_{j}{\left(Y_{\rho }\right)}_{j}\left(\sum_{i=1}^{N^{\prime }}\gamma_{i}{\left(Y_{\lambda }\right)}_{i}K_{w}\left(x_{j},x_{i}\right)\right)$$

$$=\sum_{j=1}^{N^{\prime }}\beta_{j}w_{j}{\left(Y_{\rho }\right)}_{j}{\left(T_{w}Y_{\lambda }\right)}_{j}+\sum_{j=1}^{N^{\prime }}\beta_{j}w_{j}{\left(Y_{\rho }\right)}_{j}\left(\sum_{i=1}^{N^{\prime }}\left(\gamma_{i}-\beta_{i}\right){\left(Y_{\lambda }\right)}_{i}K_{w}\left(x_{j},x_{i}\right)\right)=$$

$$={\langle Y_{\rho },T_{w}Y_{\lambda }\rangle }_{w}+\sum_{j=1}^{N^{\prime }}\beta_{j}w_{j}{\left(Y_{\rho }\right)}_{j}\left(\sum_{i=1}^{N^{\prime }}\left(\gamma_{i}-\beta_{i}\right){\left(Y_{\lambda }\right)}_{i}K_{w}\left(x_{j},x_{i}\right)\right)=$$

$$=\frac{1}{\lambda }{\langle Y_{\lambda },Y_{\rho }\rangle }_{w}+\sum_{j=1}^{N^{\prime }}\beta_{j}w_{j}{\left(Y_{\rho }\right)}_{j}\left(\sum_{i=1}^{N^{\prime }}\left(\gamma_{i}-\beta_{i}\right){\left(Y_{\lambda }\right)}_{i}K_{w}\left(x_{j},x_{i}\right)\right).$$

Finally, we obtain:

(62) $$\left(\frac{1}{\rho }-\frac{1}{\lambda }\right){\langle Y_{\lambda },Y_{\rho }\rangle }_{w}=\sum_{j=1}^{N^{\prime }}\beta_{j}w_{j}{\left(Y_{\rho }\right)}_{j}\left(\sum_{i=1}^{N^{\prime }}\left(\gamma_{i}-\beta_{i}\right){\left(Y_{\lambda }\right)}_{i}K_{w}\left(x_{j},x_{i}\right)\right).$$

Now, we shall estimate the absolute value of the expression on the left-hand side of equality (62). In the calculations below, we use the notation: ||w|| for the supremum norm of weight function *w*, $Err_{\beta }\left(\cdot \right)$ for error in the numerical integration generated by rule (51) and $Err_{\gamma }\left(\cdot \right)$ for error generated by rule (52). In addition, we normalize coordinates of eigenvectors by condition: $|{\left(Y_{\lambda }\right)}_{i}|\leq 1,i=1,$ …$,N^{\prime }$ and apply the Cauchy–Schwarz inequality:

$$\left|\left(\frac{1}{\rho }-\frac{1}{\lambda }\right){\langle Y_{\lambda },Y_{\rho }\rangle }_{w}\right|\leq$$

$$\leq \sum_{j=1}^{N^{\prime }}\beta_{j}w_{j}\left|{\left(Y_{\rho }\right)}_{j}\right|\left(\left|\sum_{i=1}^{N^{\prime }}\left(\gamma_{i}-\beta_{i}\right){\left(Y_{\lambda }\right)}_{i}K_{w}\left(x_{j},x_{i}\right)\right|\right)$$

$$\leq \sum_{j=1}^{N^{\prime }}\beta_{j}w_{j}\left|{\left(Y_{\rho }\right)}_{j}\right|\times$$

$$\times \left(\left|\sum_{i=1}^{N^{\prime }}\gamma_{i}{\left(Y_{\lambda }\right)}_{i}K_{w}\left(x_{j},x_{i}\right)\mp \int_{a}^{b}y_{\lambda }^{ap}\left(x\right)K_{w}\left(x_{j},x\right)dx-\sum_{i=1}^{N^{\prime }}\beta_{i}{\left(Y_{\lambda }\right)}_{i}K_{w}\left(x_{j},x_{i}\right)\right|\right)$$

$$\leq \sum_{j=1}^{N^{\prime }}\beta_{j}w_{j}\left|{\left(Y_{\rho }\right)}_{j}\right|\left(Err_{\gamma }(y_{\lambda }^{ap}\left(\cdot \right)K_{w}\left(x_{j},\cdot \right)+Err_{\beta }(y_{\lambda }^{ap}\left(\cdot \right)K_{w}\left(x_{j},\cdot \right)\right)$$

$$\leq 2\sum_{j=1}^{N^{\prime }}\beta_{j}w_{j}M_{err,j}\leq 2||w||\sqrt{\sum_{j=1}^{N^{\prime }}\beta_{j}^{2}}\sqrt{\sum_{j=1}^{N^{\prime }}{\left(M_{err,j}\right)}^{2}}.$$

□

## 4. Examples of Numerical Solution

Now, we shall present some numerical results of solution FSLP with homogeneous Neumann boundary conditions. We choose a very simple quadrature rule—the midpoint rectangular rule because the numerical evaluations of kernel values $K_{w}$ are very time-consuming operations. Thus, we obtain quadrature nodes ${\tilde{x}}_{i}=a+\left(i-0.5\right)\Delta x$, for i=1, …,N and $\Delta x=\frac{b-a}{N}$, and we obtain the following system of *N* linear algebraic equations corresponding to the reduced discrete integral FSLPN (50):

(63) $$y\left({\tilde{x}}_{i}\right)=\lambda T_{w}y\left({\tilde{x}}_{i}\right)\;T_{w}y\left({\tilde{x}}_{i}\right)=\frac{b-a}{N}\sum_{j=1}^{N}K_{w}\left({\tilde{x}}_{i},{\tilde{x}}_{j}\right)y\left({\tilde{x}}_{j}\right),\;i=1,\cdots ,N.$$

We write the above system of equations in the following matrix form:

(64) $$Y=\lambda T_{w}Y\;\mathrm{or}\;{\left(T_{w}\right)}^{-1}Y=\lambda Y,$$

where

(65) $$Y=\left[\begin{array}{c} y_{1}\\ y_{2}\\ \vdots \\ y_{N} \end{array}\right],\;T_{w}=\frac{b-a}{N}\left[\begin{array}{cccc} {\left(K_{w}\right)}_{1,1} & {\left(K_{w}\right)}_{1,2} & \cdots & {\left(K_{w}\right)}_{1,N}\\ {\left(K_{w}\right)}_{2,1} & {\left(K_{w}\right)}_{2,2} & & {\left(K_{w}\right)}_{2,N}\\ \vdots & & \ddots & \vdots \\ {\left(K_{w}\right)}_{N,1} & {\left(K_{w}\right)}_{N,2} & \cdots & {\left(K_{w}\right)}_{N,N} \end{array}\right]$$

while $y_{i}=y\left({\tilde{x}}_{i}\right)$ and ${\left(K_{w}\right)}_{i,j}=\;K_{w}\left({\tilde{x}}_{i},{\tilde{x}}_{j}\right),$ where

(66) $$K_{w}\left({\tilde{x}}_{i},{\tilde{x}}_{j}\right)=w\left({\tilde{x}}_{j}\right)\left(K_{1}\left({\tilde{x}}_{i},{\tilde{x}}_{j}\right)-\frac{\int_{a}^{b}w\left(\xi \right)K_{1}\left(\xi ,{\tilde{x}}_{j}\right)d\xi }{\int_{a}^{b}w\left(\xi \right)d\xi }\right)$$

with the symmetric kernel part:

(67) $$K_{1}\left({\tilde{x}}_{i},{\tilde{x}}_{j}\right)=\frac{1}{\Gamma^{2}\left(\alpha \right)}\int_{a}^{\min\left({\tilde{x}}_{i},{\tilde{x}}_{j}\right)}\frac{{\left({\tilde{x}}_{i}-t\right)}^{\alpha -1}{\left({\tilde{x}}_{j}-t\right)}^{\alpha -1}}{p\left(t\right)}dt.$$

For function w(x)=1, Equation (66) can be simplified to the form:

(68) $$K_{w}\left({\tilde{x}}_{i},{\tilde{x}}_{j}\right)=K_{1}\left({\tilde{x}}_{i},{\tilde{x}}_{j}\right)-\frac{1}{b-a}\int_{a}^{b}K_{1}\left(\xi ,{\tilde{x}}_{j}\right)d\xi .$$

The discrete/matrix eigenvalue problem (64) can be solved by using mathematical software. The resulting solution is the set of eigenvalues $\lambda_{\left(k\right)}$, for k=1, …,N and the set of eigenvectors $Y_{\lambda_{\left(k\right)}}$, for k=1, …,N, that correspond to the eigenvalues $\lambda_{\left(k\right)}$, satisfying:

(69) $$\left({\left(T_{w}\right)}^{-1}-\lambda_{\left(k\right)}\;I\right)Y_{\lambda_{\left(k\right)}}=0.$$

In order to obtain the numerical solution at all points in the considered interval $\left[a,b\right]$, we construct the approximate eigenfunctions, for example, using the step function χ:

(70) $$y_{\lambda_{\left(k\right)}}^{ap}\left(x\right)=\sum_{j=1}^{N}{\left(Y_{\lambda_{\left(k\right)}}\right)}_{j}\chi_{\left[{\tilde{x}}_{j}-e,{\tilde{x}}_{j}+e\right)}\left(x\right),\;e:=\frac{b-a}{2N}.$$

Now, we report on two examples of numerical calculations of eigenvalues and eigenfunctions to verify the proposed numerical method. In both examples, we consider the interval of calculations [0,1] and the order of derivatives α∈{1,0.8,0.6}. Representative results for the test problem are collected and presented in the form of graphs and tables. The values of $err_{\lambda }$, presented in tables, have been calculated for the fixed parameters α and variable values of *N* utilizing the following formula [48]:

(71) $$erc_{\lambda }\left(N,\alpha ,k\right)=\log_{2}\frac{\lambda_{\left(k\right)}^{\left(N,\alpha \right)}-\lambda_{\left(k\right)}^{\left(N/2,\alpha \right)}}{\lambda_{\left(k\right)}^{\left(2N,\alpha \right)}-\lambda_{\left(k\right)}^{\left(N,\alpha \right)}}.$$

Moreover, the approximate eigenfunctions were normalized by:

(72) $$\int_{a}^{b}w\left(x\right)\;y_{\lambda_{\left(k\right)}}^{ap}\left(x\right)\;y_{\lambda_{\left(k\right)}}^{ap}\left(x\right)\;dx=1,\;k=1,\cdots ,N.$$

### 4.1. Example I

**Figure 1:**
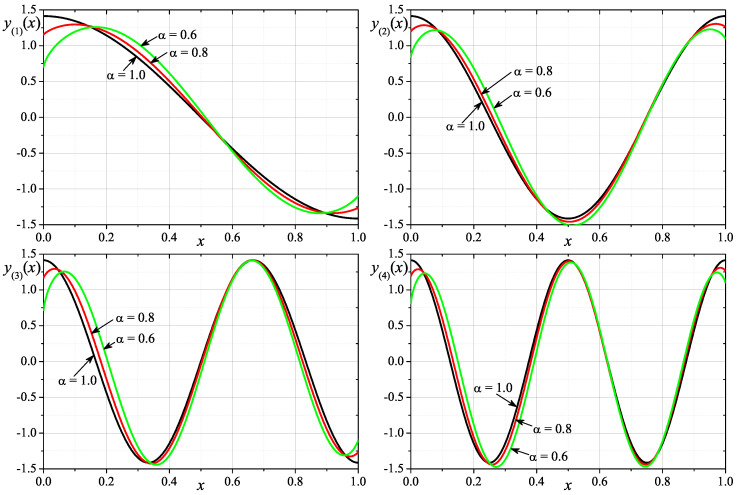
Eigenfunctions for the first 4 eigenvalues for p(x)=1, q(x)=0, w(x)=1 and α∈{1,0.8,0.6}.

The first example is devoted to the fractional equivalent of the classical harmonic oscillator problem with p(x)=1, q(x)=0 and w(x)=1. Figure 1 shows graphs of the approximate eigenfunctions corresponding to the first four eigenvalues for the considered problem. The calculations that are presented on the plot have been performed for N=4000. In Table 1, we present the numerical values of the first eight eigenvalues and the experimental rate of convergence $erc_{\lambda }$ of numerical calculations of the *k*-th eigenvalue. The table contains numerical results obtained for orders α∈{1,0.8,0.6} and different values of N∈{250,500,1000,2000,4000}.

### 4.2. Example II

**Figure 2:**
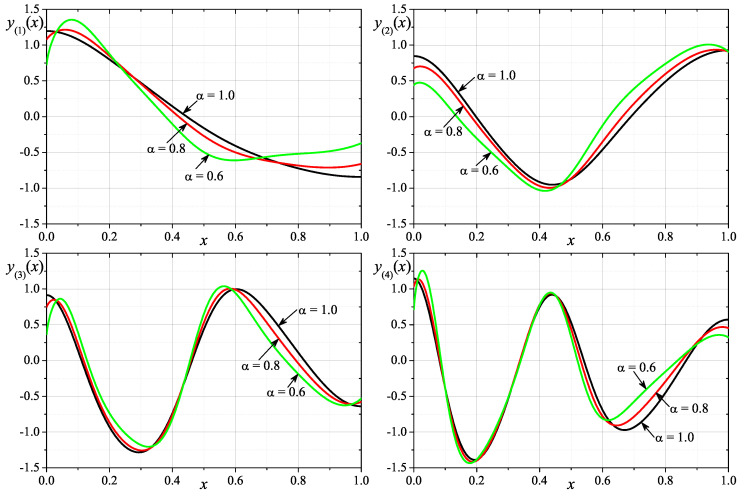
Eigenfunctions for the first 4 eigenvalues for $p\left(x\right)=2x^{2}+1$, q(x)=0 and w(x)=cos(4πx)+2 and α∈{1,0.8,0.6}.

The second example contains numerical results obtained for FSLPN with functions: $p\left(x\right)=2x^{2}+1$, q(x)=0 and w(x)=cos(4πx)+2. Figure 2 shows graphs of the approximate eigenfunctions corresponding to the first four eigenvalues for the considered problem. In this case, the calculations presented on the plot have also been performed for N=4000. In Table 2, we present the numerical values of the first eight eigenvalues and the experimental rate of convergence $erc_{\lambda }$ of numerical calculations of the *k*-th eigenvalue. The table contains the numerical results obtained for orders α∈{1,0.8,0.6} and different values of N∈{250,500,1000,2000,4000}.

## 5. Conclusions

In the paper, the FSLP with the fractional Neumann boundary conditions was studied. The considered homogeneous Neumann boundary conditions require an additional integral constraint on the solutions’ space. The additional integral condition causes the considered eigenvalue problem to become more subtle and complex than the FSLP with homogeneous Dirichlet, mixed or Robin conditions. This fact was a premise for examining the particular problem with assumption q=0. Such a problem (the differential FSLPN) was transformed to the equivalent integral one on a suitable function space. This transformation was based on results presented in the paper [51]. In the main part of the paper, the construction of the discrete version of the integral FSLPN was developed and studied. Furthermore, the orthogonality property of teh eigenvectors of the discrete integral operator was analyzed. These considerations include two particular cases of the construction of the discrete integral FSLPN. The first case is devoted to the discrete integral FSLPN received by utilizing a single numerical integration scheme. Based on such an assumption, the orthogonality (with respect to the adequate scalar product) of eigenvectors corresponding to distinct eigenvalues of the considered discrete FSLPN was proved. The second case covers the situation when the discrete integral FSLPN was derived by applying a hybrid numerical scheme (two different numerical integration schemes: one in the construction of the discrete integral FSLO and another for the integral condition restricting the solutions’ space). In this case, the orthogonality of the eigenvectors of the constructed discrete FSLPN has not been proved. However, the control of orthogonality breaking for eigenvectors of the discrete integral operator was established in Lemma 4. In the final part of the paper, two examples of numerical calculations of the eigenvalues and eigenvectors were reported. The performed calculations show that the experimental rate of convergence depends on the fractional order α and is close to 2α.

## Figures and Tables

**Table 1:** Numerical values of the first 8 eigenvalues and the experimental rates of convergence $erc_{\lambda }$ for α∈{1,0.8,0.6}, p(x)=1, q(x)=0 and w(x)=1.

		α=1		α=0.8		α=0.6	
k	N	$\lambda_{\left(k\right)}$	${\mathrm{erc}}_{\lambda }$	$\lambda_{\left(k\right)}$	${\mathrm{erc}}_{\lambda }$	$\lambda_{\left(k\right)}$	${\mathrm{erc}}_{\lambda }$
1	250	9.8694745	-	7.8556424	-	5.4750947	-
	500	9.8695719	1.997	7.8571537	1.610	5.5029938	1.191
	1000	9.8695963	2.000	7.8576488	1.607	5.5152125	1.197
	2000	9.8696024	2.024	7.8578113	1.608	5.5205439	1.199
	4000	9.8696039	-	7.8578646	-	5.5228666	-
2	250	39.476340	-	20.327286	-	10.008656	-
	500	39.477898	1.998	20.337158	1.602	10.102136	1.182
	1000	39.478288	2.007	20.340410	1.602	10.143348	1.192
	2000	39.478385	2.015	20.341481	1.601	10.161385	1.197
	4000	39.478409	-	20.341834	-	10.169253	-
3	250	88.815920	-	38.881625	-	16.167499	-
	500	88.823810	2.000	38.917945	1.603	16.412925	1.170
	1000	88.825782	2.000	38.929901	1.603	16.522008	1.187
	2000	88.826275	1.991	38.933836	1.603	16.569919	1.195
	4000	88.826399	-	38.935131	-	16.590853	-
4	250	157.88042	-	59.665008	-	21.512600	-
	500	157.90536	2.001	59.749754	1.599	21.948949	1.159
	1000	157.91159	1.998	59.777723	1.601	22.144364	1.182
	2000	157.91315	2.000	59.786945	1.601	22.230487	1.192
	4000	157.91354	-	59.789984	-	22.268175	-
5	250	246.65895	-	85.292837	-	27.925213	-
	500	246.71982	2.000	85.466870	1.600	28.665274	1.147
	1000	246.73504	2.002	85.524297	1.602	28.999570	1.177
	2000	246.73884	2.000	85.543221	1.602	29.147466	1.190
	4000	246.73979	-	85.549456	-	29.212295	-
6	250	355.13747	-	112.54371	-	33.623633	-
	500	355.26368	2.000	112.84505	1.596	34.701683	1.135
	1000	355.29524	2.000	112.94470	1.600	35.192608	1.171
	2000	355.30313	2.002	112.97757	1.599	35.410598	1.187
	4000	355.30510	-	112.98842	-	35.506312	-
7	250	483.29886	-	144.00145	-	40.125471	-
	500	483.53266	2.000	144.49694	1.596	41.671012	1.122
	1000	483.59113	2.001	144.66082	1.600	42.381118	1.165
	2000	483.60574	1.997	144.71487	1.601	42.697703	1.185
	4000	483.60940	-	144.73269	-	42.836953	-
8	250	631.12288	-	176.75038	-	45.983903	-
	500	631.52170	2.000	177.49404	1.594	48.024089	1.110
	1000	631.62143	2.000	177.74045	1.598	48.969362	1.160
	2000	631.64637	2.001	177.82183	1.600	49.392428	1.182
	4000	631.65260	-	177.84867	-	49.578838	-

**Table 2:** Numerical values of the first 8 eigenvalues and the experimental rates of convergence $erc_{\lambda }$ for α∈{1,0.8,0.6}, $p\left(x\right)=2x^{2}+1$, q(x)=0 and w(x)=cos(4πx)+2.

		α=1		α=0.8		α=0.6	
k	N	$\lambda_{\left(k\right)}$	${\mathrm{erc}}_{\lambda }$	$\lambda_{\left(k\right)}$	${\mathrm{erc}}_{\lambda }$	$\lambda_{\left(k\right)}$	${\mathrm{erc}}_{\lambda }$
1	250	7.0687342	-	6.2956345	-	4.6051127	-
	500	7.0687782	2.000	6.2967500	1.622	4.6361847	1.195
	1000	7.0687892	1.974	6.2971124	1.620	4.6497610	1.199
	2000	7.0687920	2.000	6.2972303	1.615	4.6556764	1.200
	4000	7.0687927	-	6.2972688	-	4.6582511	-
2	250	24.644959	-	12.771392	-	6.4333679	-
	500	24.646018	1.999	12.777978	1.602	6.4923883	1.181
	1000	24.646283	2.005	12.780147	1.603	6.5184265	1.192
	2000	24.646349	1.957	12.780861	1.597	6.5298259	1.197
	4000	24.646366	-	12.781097	-	6.5347998	-
3	250	70.274276	-	30.075510	-	12.136357	-
	500	70.279914	1.999	30.101699	1.604	12.331176	1.174
	1000	70.281324	2.002	30.110313	1.604	12.417505	1.189
	2000	70.281676	2.000	30.113146	1.604	12.455371	1.195
	4000	70.281764	-	30.114078	-	12.471906	-
4	250	127.27968	-	46.851483	-	16.228206	-
	500	127.29583	1.999	46.912533	1.602	16.596768	1.171
	1000	127.29987	2.000	46.932645	1.603	16.760495	1.187
	2000	127.30088	2.014	46.939266	1.603	16.832387	1.195
	4000	127.30113	-	46.941446	-	16.863793	-
5	250	197.81960	-	67.232260	-	20.906261	-
	500	197.88521	1.999	67.418158	1.600	21.637418	1.143
	1000	197.90162	2.001	67.479487	1.602	21.968596	1.175
	2000	197.90572	2.007	67.499697	1.602	22.115285	1.189
	4000	197.90674	-	67.506354	-	22.179616	-
6	250	285.02794	-	89.803850	-	25.836658	-
	500	285.14300	2.000	90.081339	1.598	26.887072	1.144
	1000	285.17177	2.001	90.173017	1.600	27.362312	1.175
	2000	285.17896	1.998	90.203255	1.601	27.572716	1.189
	4000	285.18076	-	90.213224	-	27.664975	-
7	250	381.52715	-	112.94393	-	30.374162	-
	500	381.75312	2.000	113.43053	1.598	31.911832	1.117
	1000	381.80962	2.001	113.59131	1.601	32.620555	1.164
	2000	381.82374	1.996	113.64431	1.601	32.936757	1.185
	4000	381.82728	-	113.66178	-	33.075861	-
8	250	495.02316	-	136.38509	-	34.838979	-
	500	495.40389	2.000	137.06049	1.593	36.579470	1.111
	1000	495.49909	2.000	137.28436	1.598	37.385104	1.159
	2000	495.52289	2.000	137.35829	1.600	37.745787	1.182
	4000	495.52884	-	137.38267	-	37.904751	-

## Data Availability

Not applicable.
